# Supplementary material for: Evaluation of Knowledge and Risk Perception about Antibiotic Resistance in Biology and Mathematics Young Students in Nîmes University in France
Source: Int J Environ Res Public Health. 2021 Sep 14;18(18):9692. doi: 10.3390/ijerph18189692 (PMC8468539; doi:10.3390/ijerph18189692)
Supplement: Supplementary file 1 [file ijerph-18-09692-s001.zip › Additional file 1.pdf]

- Supporting Information –

Evaluation of Knowledge and Risk Perception about Antibiotic Resistance of Biology and Mathematics Young Students in Nîmes University in France

Valentin Duvauchelle,[1] Elsa Causse,[1#] Julien Michon,[1] Patrick Rateau,[2] Karine Weiss,[1] Patrick Meffre[1] and Zohra Benfodda[1*#]

***Correspondence**: Dr. Zohra Benfodda, University of Nîmes, CHROME laboratory, EA7352, Rue du Dr. G. Salan, 30021 Nîmes Cedex 1, France. Email: zohra.benfodda@unimes.fr

Table of contents

Figure S1. Questionnaire passed in English

Figure S2. Questionnaire passed in French

Figure S1. Questionnaire passed in English

QUESTIONNAIRE

Age: Gender: ☐ Female ☐ Male

Degree course: Year of Study: ☐ 1st ☐ 2nd ☐ 3rd

Please read the statements carefully and answer the following questions. There are no right or wrong answers, only your opinion and experience counts. Your answers will be treated anonymously. Thank you in advance for your participation.

1) When was the last time you took antibiotics?
- ☐ Within the last month
- ☐ In the last six months
- ☐ Over the past year
- ☐ More than a year ago
- ☐ Never
- ☐ I can't remember

If the "Never" box has been checked, go directly to question 4.

2) Were you prescribed antibiotics by a doctor at that time?
- ☐ Yes
- ☐ No
- ☐ I can't remember

3) On this occasion, did you receive any indications concerning the taking of antibiotics (before or after a meal, 7-day period...) from a health professional (doctor, pharmacist or nurse)?
- ☐ Yes
- ☐ No
- ☐ I don't remember

4) After starting treatment, when do you think you should stop taking antibiotics?
- ☐ When you feel better
- ☐ When you've taken all the antibiotics as directed
- ☐ I don't know

5) Do you think that antibiotics that have been given to others (a friend or family member) can be used as long as they have been used to treat the same illness?
- ☐ I think it's possible
- ☐ I don't think it's possible
- ☐ I don't know

6) Do you think you can ask a doctor for the same antibiotics, if they have treated the same symptoms in the past?
- ☐ I think it's possible
- ☐ I don't think it's possible
- ☐ I don't know

7) Which of the following diseases/disorders can be treated with antibiotics (multiple choice):
- ☐ HIV/AIDS
- ☐ Gonorrhea
- ☐ Bladder infection or urinary tract infection
- ☐ Diarrhea
- ☐ Influenza
- ☐ Fever
- ☐ Malaria
- ☐ Measles
- ☐ Skin infection
- ☐ Traumatic wound
- ☐ Sore throat
- ☐ Body aches
- ☐ Headaches

8) Below is a list of statements, please express your opinion on each of these sentences by assigning a "score" ranging from 1 = "not agree at all" to 6 = "completely agree". There are no right or wrong answers, only your opinion.

	Not agree at all<br>1	2	3	4	5	Completely agree<br>6
1. Many infections are becoming increasingly resistant to antibiotic treatment.						
2. Antibiotic resistance occurs when the body becomes resistant to antibiotics.						
3. People should use antibiotics only when prescribed by their doctor.						
4. Antibiotic resistance is a problem that can affect me or my family.						
5. Antibiotic resistance can be partly caused by self-medication.						
6. Antibiotic resistance is a problem in other countries, but not in France.						
7. Antibiotic resistance is only a problem for those who take antibiotics regularly.						
8. Bacteria that are resistant to antibiotics are not transmitted from person to person.						
9. Antibiotic-resistant infections can make medical procedures such as surgery, organ transplants or cancer treatments more dangerous.						
10. In France, antibiotics are not used in animal husbandry.						
11. Antibiotic resistance is one of the biggest problems facing the world.						
12. Doctors should prescribe antibiotics only when necessary.						
13. If bacteria become resistant to antibiotics, it becomes very difficult or impossible to treat the infections they cause.						
14. People like me can't do anything about antibiotic resistance.						
15. I am not at risk of getting an antibiotic resistant infection as long as I take my antibiotics correctly.						
16. Washing my hands decreases the spread of resistance.						
17. Livestock producers have little involvement in the spread of bacterial resistance.						
18. I am not concerned about the impact of bacterial resistance on my health or that of my family.						
19. Resistant bacteria spread resistance to each other.						
20. I can take antibiotics with tea or coffee.						

9) Now answer the following questions by circling the number that most closely corresponds to your personal opinion (answers ranging from 1 = not agree at all to 10 = completely agree).

1. I consider antibiotic resistance to be a risk.

Not agree at all	1	2	3	4	5	6	7	8	9	10	Completely agree

2. I am concerned about this risk (antibiotic resistance).

Not agree at all	1	2	3	4	5	6	7	8	9	10	Completely agree

3. I consider myself particularly well informed about this risk (antibiotic resistance).

Not agree at all	1	2	3	4	5	6	7	8	9	10	Completely agree

4. Scientists have a very large knowledge of this risk (antibiotic resistance).

Not agree at all	1	2	3	4	5	6	7	8	9	10	Completely agree

5. I consider that antibiotic resistance represents a new risk.

Not agree at all	1	2	3	4	5	6	7	8	9	10	Completely agree

6. Science is aware of all the effects of this risk (antibiotic resistance) on human health.

Not agree at all	1	2	3	4	5	6	7	8	9	10	Completely agree

7. New and still No risks detected today regarding antibiotic resistance will be discovered in the future.

Not agree at all	1	2	3	4	5	6	7	8	9	10	Completely agree

8. I feel that I can do something to avoid this risk (antibiotic resistance).

Not agree at all	1	2	3	4	5	6	7	8	9	10	Completely agree

Figure S2. Questionnaire passed in French

QUESTIONNAIRE

Âge : **Genre : ☐ Féminin ☐ Masculin**

Cursus : **Année d'étude : ☐ 1ère ☐ 2ème ☐ 3ème**

Veuillez lire attentivement les énoncés et répondre aux questions qui suivent. Il n'y a pas de bonne ou de mauvaise réponse, seul votre avis et votre expérience comptent. Vos réponses seront traitées de façon anonyme. Merci par avance pour votre participation.

1) **Quand avez-vous pris des antibiotiques pour la dernière fois ?**
   - ☐ Au cours du dernier mois
   - ☐ Au cours des six derniers mois
   - ☐ Au cours de la dernière année
   - ☐ Il y a plus d'une année
   - ☐ Jamais
   - ☐ Je ne m'en souviens pas

Si la case « Jamais » a été cochée, allez directement à la question 4.

2) **À cette occasion, les antibiotiques vous ont-ils été prescrits par un médecin ?**
   - ☐ Oui
   - ☐ Non
   - ☐ Je ne m'en souviens pas
3) **À cette occasion, avez-vous reçu des indications concernant la prise des antibiotiques (avant ou après le repas, période de 7 jours…) de la part d'un professionnel de la santé (médecin, pharmacien ou infirmier) ?**
   - ☐ Oui
   - ☐ Non
   - ☐ Je ne m'en souviens pas
4) **Après le début du traitement, à quel moment pensez-vous qu'il faille arrêter la prise d'antibiotiques ?**
   - ☐ Quand on se sent mieux
   - ☐ Quand on a pris tous les antibiotiques prescrits
   - ☐ Je ne sais pas
5) **Pensez-vous que l'on puisse utiliser les antibiotiques qui ont été donnés à d'autres personnes (un ami ou un membre de sa famille), du moment qu'ils ont été utilisés pour soigner la même maladie ?**
   - ☐ Je pense que c'est possible
   - ☐ Je pense que ce n'est pas possible
   - ☐ Je ne sais pas

6) **Pensez-vous que l'on puisse demander les mêmes antibiotiques à un médecin, s'ils ont permis de traiter les mêmes symptômes par le passé ?**
   - ☐ Je pense que c'est possible
   - ☐ Je pense que ce n'est pas possible
   - ☐ Je ne sais pas

7) **Quelles maladies/troubles parmi ceux ci-dessous est-il possible de traiter à l'aide d'antibiotiques (choix multiples) :**
   - ☐ HIV/SIDA
   - ☐ Gonorrhée
   - ☐ Infection vessie/urinaire
   - ☐ Diarrhée
   - ☐ Grippe
   - ☐ Fièvre
   - ☐ Malaria
   - ☐ Rougeole
   - ☐ Infection de la peau
   - ☐ Plaie traumatique
   - ☐ Mal de gorge
   - ☐ Courbatures

☐ Mal de tête

8) Voici une liste d'affirmations, veuillez exprimer votre opinion par rapport à chacune de ces phrases en attribuant une « note » allant de 1 = « pas du tout d'accord » à 6 = « tout à fait d'accord ». Il n'y a pas de bonne ou de mauvaise réponse, seul votre avis nous intéresse.

	**Pas du tout d'accord 1**	**2**	**3**	**4**	**5**	**Tout à fait d'accord 6**
1. De nombreuses infections deviennent de plus en plus résistantes aux traitements antibiotiques.						
2. La résistance aux antibiotiques intervient quand le corps devient résistant aux antibiotiques.						
3. Les gens devraient utiliser les antibiotiques seulement lorsqu'ils sont prescrits par leur médecin.						
4. La résistance aux antibiotiques est un problème qui peut me toucher moi ou ma famille.						
5. La résistance aux antibiotiques peut être en partie causée par l'automédication.						
6. La résistance aux antibiotiques est un problème dans les autres pays, mais pas en France.						
7. La résistance aux antibiotiques est seulement un problème pour ceux qui prennent régulièrement des antibiotiques.						
8. Les bactéries qui sont résistantes aux antibiotiques ne se transmettent pas de personne en personne.						
9. Les infections résistantes aux antibiotiques peuvent rendre plus dangereuses les procédures médicales comme la chirurgie, transplantations d'organes ou traitements de cancers.						
10. En France, les antibiotiques ne sont pas utilisés dans l'élevage.						
11. La résistance aux antibiotiques est un des plus gros problèmes auquel le monde fait face.						
12. Les docteurs devraient prescrire les antibiotiques uniquement lorsque cela est nécessaire.						
13. Si les bactéries deviennent résistantes aux antibiotiques, il devient très difficile voire impossible de traiter les infections qu'elles entraînent.						
14. Les personnes comme moi ne peuvent rien faire pour lutter contre la résistance aux antibiotiques.						
15. Je ne risque pas de contracter une infection résistante aux antibiotiques tant que je prends mes antibiotiques correctement.						
16. Se laver les mains diminue la propagation des résistances.						
17. Les éleveurs de bétail participent peu au phénomène de résistance bactérienne.						
18. Je ne suis pas inquiet quant à l'impact de la résistance bactérienne sur ma santé ou celle de ma famille.						
19. Les bactéries résistantes se transmettent les résistances entre elles.						
20. Je peux prendre des antibiotiques avec du thé ou du café.						

9) Répondez maintenant aux propositions suivantes en entourant le chiffre correspondant le plus à votre avis personnel (réponses allant de 1 = pas du tout d'accord à 10 = tout à fait d'accord).

1. Je considère que la résistance aux antibiotiques représente un risque.

Pas du tout d'accord	1	2	3	4	5	6	7	8	9	10	Tout à fait d'accord

2. Je suis préoccupé par ce risque (résistance aux antibiotiques).

Pas du tout d'accord	1	2	3	4	5	6	7	8	9	10	Tout à fait d'accord

3. Je m'estime particulièrement bien informé sur ce risque (résistance aux antibiotiques).

Pas du tout d'accord	1	2	3	4	5	6	7	8	9	10	Tout à fait d'accord

4. Les scientifiques ont une très grande connaissance de ce risque (résistance aux antibiotiques).

Pas du tout d'accord	1	2	3	4	5	6	7	8	9	10	Tout à fait d'accord

5. Je considère que la résistance aux antibiotiques représente un risque nouveau.

Pas du tout d'accord	1	2	3	4	5	6	7	8	9	10	Tout à fait d'accord

6. La science connait, concernant ce risque (résistance aux antibiotiques), tous ses effets sur la santé humaine.

Pas du tout d'accord	1	2	3	4	5	6	7	8	9	10	Tout à fait d'accord

7. Des risques nouveaux et encore non décelés aujourd'hui concernant la résistance aux antibiotiques vont être découverts à l'avenir.

Pas du tout d'accord	1	2	3	4	5	6	7	8	9	10	Tout à fait d'accord

8. J'ai le sentiment de pouvoir agir pour éviter ce risque (résistance aux antibiotiques).

Pas du tout d'accord	1	2	3	4	5	6	7	8	9	10	Tout à fait d'accord
